# Early right ventricular dysfunction after transcatheter aortic valve replacement (TAVI): a prospective cardiac magnetic resonance study of open versus transcatheter aortic valve replacement

**DOI:** 10.1186/1532-429X-15-S1-P278

**Published:** 2013-01-30

**Authors:** Gareth Crouch, Jayme Bennetts, Ajay Sinhal, Craig Bradbrook, Robert A Baker, Joseph Selvanayagam

**Affiliations:** 1 Cardiovascular Medicine, Flinders Medical Centre, Adelaide, SA, Australia; 2 Cardiothoracic Surgery, Flinders Medical Centre, Adelaide, SA, Australia; 3 Flinders Centre for Cardiovascular Magnetic Resonance Research, Flinders University, Adelaide, SA, Australia

## Background

Recent published literature has validated the use of transcatheter aortic valve implantation (TAVI) in high-risk patients with aortic stenosis. These trials and registries have largely focused on combined morbidity and mortality outcomes with little focus given to impact on early myocardial function. We assessed effects on myocardial function, reversible and irreversible myocardial injury of both transcatheter and open aortic valve replacement utilizing multi-parametric CMR.

## Methods

We conducted a prospective comparative study of 38 patients (20 male) with confirmed severe aortic stenosis undergoing either transcatheter valve replacement (20 patients) or high risk (euroSCORE >12) open AVR. CMR was carried out pre-operatively and within two weeks post-operatively. All scans used a Siemens Aera 1.5T system (Siemens, Germany). ). Images obtained included a standard LV long and short axis SSFP imaging, T2 weighted images using LV basal, mid and apical SA slices and late gadolinium enhanced (LGE) images (Gadovist 0.1mg/kg) in standard LV long and SA planes.

## Results

There was no difference in log euroSCORE's between the groups pre-operatively. Post-operative CMR was conducted at a median of 6.0 days for TAVI and 7.3 days for Open (p>0.05). Mean preoperative LV ejection fraction (LVEF) and right ventricular ejection fraction (RVEF) was similar in the 2 groups (LVEF TAVI 65.3% vs Open 70.5%, RVEF TAVI 58.3% vs 58.1%). Post-operatively LVEF was preserved in both groups. In contrast, RVEF decreased significantly in the TAVI group when compared to the open group (58.3% to 49.4% vs 58.1% to 56.0% p= 0.02). This was largely driven by reduced RVESV in the open group and increased RVESV in the TAVI group (p = 0.04). The open AVR group demonstrated a trend towards increased early LV mass regression compared to the TAVI group (p = 0.08). Myocardial injury assessed biochemically using HS troponin and area under the curve showed significantly greater injury in the open cohort (p=0.001). A total of 3 patients in TAVI group and 2 patients in open AVR group demonstrated new LV irreversible injury (p>0.05).

## Conclusions

There is no significant difference in early left ventricular functional outcomes between Open and transcatheter techniques despite higher troponin in the open cohort. In the absence of new RV irreversible myocardial injury, it is likely that the RV dysfunction seen in the TAVI group is a result of rapid ventricular pacing during device insertion, resulting in myocardial stunning.

## Funding

Research grant from Flinders Cardiothoracic Research Fund.

